# Discovery of potential genes contributing to the biosynthesis of short-chain fatty acids and lactate in gut microbiota from systematic investigation in *E. coli*

**DOI:** 10.1038/s41522-019-0092-7

**Published:** 2019-07-12

**Authors:** Chunhua Zhao, Hongjun Dong, Yanping Zhang, Yin Li

**Affiliations:** 1 CAS Key Laboratory of Microbial Physiological and Metabolic Engineering, State Key Laboratory of Microbial Resources, Institute of Microbiology, Chinese Academy of Sciences, Beijing, China; 2 University of Chinese Academy of Sciences, Beijing, China; 3 Present Address: Department of Chemistry, University of California, Berkeley, Berkeley, CA USA

**Keywords:** Microbial communities, Applied microbiology, Bacteria

## Abstract

Microbiota play important roles in the internal environment and health of humans, livestock and wild animals. Short-chain fatty acids (SCFAs) and lactate are primary metabolites that can impact the composition and function of human microbiota. According to the well-characterized key synthesis genes, many SCFA- and lactate-producing bacteria have been identified in the gut microbiota. However, unknown genes may also contribute to the formation of SCFAs and lactate. The identification of such genes will provide new engineering targets and new strategies for maintaining a stable structure of beneficial microbiota. In this study, we used *Escherichia coli* as a model to analyze possible genes related to SCFAs and lactate production besides the well-characterized ones. The functions of nineteen candidate genes were studied by targeted gene deletion and overexpression. Results indicated thioesterase genes such as *yciA*, *tesA*, *tesB*, and *menI* can contribute to acetate and/or butyrate formation. As for lactate, *mgsA* and *lldD* can function in addition to *ldh* gene. At the same time, the distribution of these functional genes in gut microbiota was investigated. Most bacteria contain the well-studied genes whereas some bacteria contain some of the described unusual ones. The results provide insights and genetic targets for the discovery of new SCFA- and lactate-producing bacteria in gut microbiota.

## Introduction

**Fig. 1:**
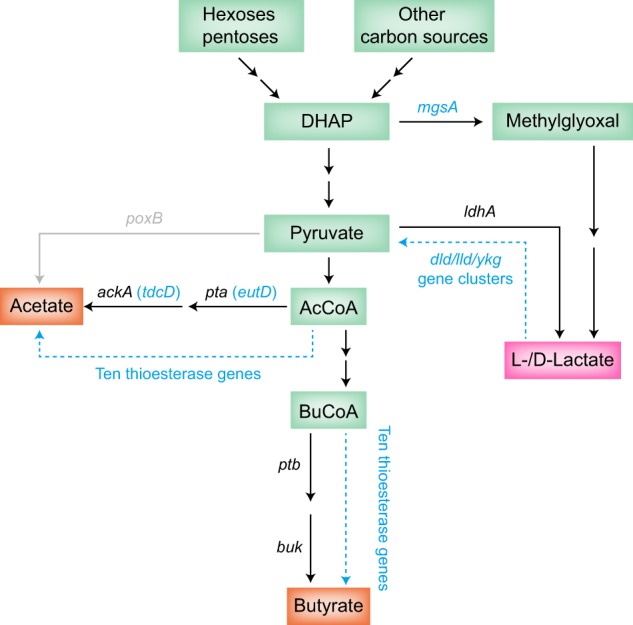
Synthetic pathways of SCFAs and lactate in bacteria. The genes shown in blue or dashed line are described in this paper. Most genes were studied in *E. coli* strains. The *ptb*-*buk* gene does not exist in *E. coli* but in some *Clostridium* strains. DHAP: dihydroxyacetone-phosphate; AcCoA: acetyl-CoA; BuCoA: butyryl-CoA

Gut microbiota have been shown to play important roles in the intestinal and even general health of humans and livestock.^1,2^ The complex composition of bacteria in the intestinal tract make the overall metabolism in the intestinal environment quite complex.^2^ However, within this complexity, there are a small number of general metabolic modes. In healthy adults, incompletely digested food and other components are anaerobically fermented to produce mainly gases and organic acids for ATP and redox homeostasis demands. Short-chain fatty acids (SCFAs), including acetate, propionate, and butyrate, are the main organic acids present in the intestines,^2,3^ and were proved to be beneficial for human health.^4–6^ While acetate is generally derived from pyruvate or acetyl-CoA,^7^ it can also be formed by acetogens which employ the Wood-Ljungdahl pathway.^2,8^ It is known that acetate production from pyruvate or acetyl-CoA is mainly catalyzed by phosphate acetyltransferase (encoded by *pta*) and acetate kinase (encoded by *ackA*).^7^ Propionate is mostly produced via the succinate pathway, but can also be derived from the acrylate and propanediol pathways.^2,9^ Butyrate is mainly derived from butyryl-CoA.^2,10^ Similarly to acetate, butyrate is produced via the action of phosphate butyryltransferase (encoded by *ptb*) and butyrate kinase (encoded by *buk*).^11^ In addition to the common SCFAs, lactate is easily produced by intestinal lactic acid bacteria (LAB), bifidobacteria, and other anaerobes.^12–14^ Lactate dehydrogenase (encoded by *ldh*) is the major contributor to lactate production (Fig. 1).

The available information on the key genes and enzymes of SCFAs production was used to identify SCFA-producing bacteria with potential applications for host health, such as anti-inflammatory effect and energy metabolism improvement.^2,14–17^ However, yet to be discovered microbiota may also contribute to SCFAs (or lactate) production, possibly employing pathways other than the well-studied ones. Therefore, a systematic investigation of the SCFA-producing pathways may provide further insights into the metabolic complexity of gut microbiota and form a basis for finding more SCFA-producing commensal and probiotic bacteria to benefit host health.

Since *Escherichia coli* is a model microorganism and a common bacterium among the gut microbiota,^18^ we used it as a model bacterium to discover uncommon pathways contributing to the production of SCFAs and lactate. Nineteen genes possibly related to production of SCFAs and lactate were identified in the annotated genome (Fig. 1). These genes were individually inactivated to investigate the consequent effects on the quantities of different SCFAs and lactate produced. In addition, overexpression of these genes in the selected defective strain were performed. The results provide new genes which can be used to search for potential bacteria capable of producing SCFAs or lactate in the human intestines and provide new control targets for regulating the production of SCFAs or lactate by gut microbiota.

## Results

### Selection of nineteen candidate genes contributing to SCFAs and lactate production in *E. coli*

Thioesterases could function for the production of medium- and long-chain fatty acids, such as hexanoate, octoate, and laurate in *E. coli*.^19–22^ It was reported that the thioesterases were not acyl-CoA-specific, with some capable of converting the relevant acyl-CoA into SCFAs naturally.^20^ Based on this, we scanned the *E. coli* genome and identified 10 genes (*fadM*, *tesB*, *tesA*, *entH*, *ybgC*, *ybhC*, *yciA*, *paaI*, *menI*, and *yigI*) with possible thioesterase function in KEGG database.^23^ The ten thioesterase genes, along with the anaerobic phosphate acetyltransferase gene *eutD* and the acetate kinase gene *tdcD*, were chosen as target genes to examine their contributions to acetate and butyrate production in *E. coli*.

Besides the well-known NAD^+^-dependent lactate dehydrogenase (LDH, encoded by *ldhA*), some other LDH enzymes were reported to have a role in lactate production/utilization. These include an FMN-linked LDH (encoded by *lldD*), an FAD-binding d-LDH (encoded by *dld*), and an LDH complex protein (encoded by *ykgF*).^24,25^ To identify more alternative genes that may contribute to lactate synthesis, we combined database (KEGG) mining and literature searches using “lactate (or lactate production)” as key words,^24–26^ and we found that *lldD* is part of the *lldPRD* operon while *ykgF* is located within the *ykgEFG* gene cluster. Hence, all these genes, with the exception of *lldP*, which encodes lactate permease, were chosen as targets to test their contributions to lactate production when the main *ldhA*-based pathway was blocked. In addition, differing from the LDH-based pathway, there is another pathway for lactate production. The methylglyoxal synthase encoded by *mgsA* gene, which is involved in the methylglyoxal bypass, can convert dihydroxyacetone-phosphate into methylglyoxal. Methylglyoxal can be further oxidized to yield lactate by glyoxalase or aldehyde dehydrogenase.^24^ Hence, we selected a total of seven genes (*mgsA*, *dld*, *lldD*, *lldR*, *ykgE*, *ykgF*, and *ykgG*) which may have roles in lactate production.

### Identification of the contributions of twelve candidate genes to SCFAs synthesis through gene deletion

Wild-type *E. coli* produces SCFAs and lactate for energy and reducing power needs.^27,28^ In our starting strain *E. coli* EB228, the well-known genes *pta* and *ackA* (involved in acetate production) and *ldhA* (involved in lactate production) were deleted. However, it still produced 170 mg/L of acetate, 590 mg/L of butyrate, and 380 mg/L of lactate after 72 h in tube fermentations (Table 1), indicating the presence of unknown enzymes and pathways contributing to the production of SCFAs and lactate.

**Table 1:** The metabolic product profile of strain EB228

Product	Pyruvate	Lactate	Acetate	Butyrate	Ethanol	Butanol
Titer (mg/L)	130	380	170	590	410	3550

**Fig. 2:**
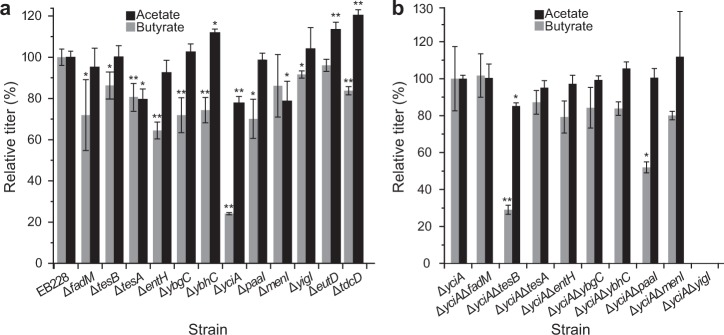
SCFAs production after the first- and the second-round of gene deletions in *E. coli*. **a** first-round deletion, the acetate and butyrate titers of strain EB228 were designated as 100%; **b** second-round deletion, the acetate and butyrate titers of strain EB228Δ*yciA* were designated as 100%. Strain EB228Δ*yciA*Δ*yigI* grew poorly in M9Y medium, reaching a final OD_600_ of only 0.5. The data represent the means ± s.d. from three biological replicates. *Statistically significant difference with *P* < 0.05 and **Statistically significant difference with *P* < 0.01

Based on strain EB228, two acetate-related genes that function under anaerobic conditions and 10 thioesterase genes were individually knocked out. The twelve mutants obtained in the first round of deletion showed different changes of acetate production (Fig. 2a). Strains EB228Δ*tesA*, EB228Δ*yciA*, and EB228Δ*menI* showed significant decrease of acetate production by 20%, 23%, and 21%, respectively. The acetate production of all other mutants did not significantly decrease. In fact, some mutants even showed an increased yield of acetate. Eleven mutants showed similar cell growth with strain EB228 while strain EB228Δ*yciA* grew better than the others (Supplementary Fig. 1a, *P* < 0.01). Interestingly, in addition to the reduction of acetate titers, butyrate production decreased even more significantly. Most of the twelve mutants exhibited reduction of butyrate titer, among which *tesA*-, *entH*-, *ybgC*-, *ybhC*-, and *yciA*-deleted strains showed highly significant changes. The sum of the related percentages was beyond 100%, indicating that some of these genes may function synergistically in the synthesis of butyrate. This also indicates the complex biochemical networks and their regulation within living cells.

Remarkably, the *yciA* deletion strain produced the smallest yields of both acetate and butyrate. The acetate and butyrate production of strain EB228Δ*yciA* decreased by 23.5% and 77%, respectively, compared to that of the control strain EB228. This suggests that the *yciA*-encoded thioesterase can hydrolyze both acetyl-CoA and butyryl-CoA, while the latter is the preferential substrate. To test this hypothesis, we determined the specific thioesterase activity of strains EB228 and EB228Δ*yciA* using acetyl-CoA and butyryl-CoA as substrates. As shown in Table 2, after deletion of *yciA*, the specific thioesterase activity towards acetyl-CoA and butyryl-CoA decreased by 9% (*P* > 0.05) and 30% (*P* < 0.01), respectively, indicating that the *yciA*-encoded thioesterase preferentially acts on butyryl-CoA. This gene therefore represents a new target that may be used to regulate the proportions of different SCFAs.

**Table 2:** Specific thioesterase activity (nmol/min/mg protein) of EB228 and EB228Δ*yciA* toward acetyl-CoA and butyryl-CoA

Substrate	Specific thioesterase activity (nmol/min/mg protein)		Decrease by knockout of *yciA*
	EB228	EB228Δ*yciA*	
Acetyl-CoA	3.3 ± 0.3	3.0 ± 0.5	9%
Butyryl-CoA	5.0 ± 0.3	3.5 ± 0.6	30%

To investigate whether other hypothetic thioesterase-encoding genes have synergistic interactions with the *yciA* gene in SCFAs production, we did a second-round of thioesterase gene deletions based on strain EB228Δ*yciA*. Acetate production of strain EB228Δ*yciA*Δ*tesB* decreased by 15% compared to that of EB228Δ*yciA*, while it remained unaltered in the other mutants (Fig. 2b). Notably, the combinational knockout based on EB228Δ*yciA* did not decrease acetate accumulation even in the case of the two functional genes *tesA* and *menI*. This suggested that these enzymes have no synergistic action in the synthesis of acetate. By contrast, the two mutants EB228Δ*yciA*Δ*tesB* and EB228Δ*yciA*Δ*paaI* showed further significant decreases of butyrate production (Fig. 2b). Strikingly, the butyrate titer of strain EB228Δ*yciA*Δ*tesB* dropped to 50 mg/L—a 92% decrease compared to the starting strain EB228. This strain also grew better (Supplementary Fig. 1b, *P* < 0.01), which suggested that even low-level acid production could affect the cell growth. Interestingly, when only *tesB* was deleted, the butyrate production only changed a little. Strain EB228Δ*yciA*Δ*paaI* produced 90 mg/L butyrate, representing an 85% decrease compared to the starting strain EB228. In conclusion, through gene deletion, we identified three genes (*menI*, *tesA*, and *yciA*) contributing to acetate production and five genes (*entH*, *tesA*, *ybgC*, *ybhC*, and *yciA*) contributing to butyrate production.

### Identification of the contributions of ten candidate genes to SCFAs synthesis through gene overexpression

The function of a gene may be identified by gene deletion. However, some other genes may function to cover the deleted gene and restore the metabolism.^28^ Under this circumstances, gene overexpression is another approved strategy to confirm the function of a gene.

**Fig. 3:**
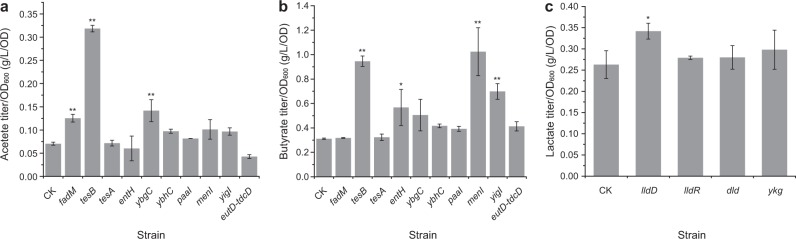
Acetate, butyrate, and lactate production per unit cell after overexpressing the related genes. **a**, **b** acetate and butyrate production. CK: EB228Δ*yciA*,pAC2; Gene name indicating strain with overexpressed corresponding gene. For instance, *fadM* indicating EB228Δ*yciA*,pAC2-*fadM*. **c** lactate production. CK: EB228Δ*mgsA*,pAC2; Gene name indicating strain with overexpressed corresponding gene. For instance, *lldD* indicating EB228Δ*mgsA*,pAC2-*lldD*. The data represent the means ± s.d. from three biological replicates. *Statistically significant difference with *P* < 0.05 and **Statistically significant difference with *P* < 0.01

Based on strain EB228Δ*yciA*, the rest nine thioesterase genes and *eutD-tdcD* (anaerobic acetate-producing genes) were cloned into vector for overexpression. As expected, the strains showed different cell growth (Supplementary Fig. 2a). To make them comparable, the SCFAs titers were standardized by the OD_600_ values. For acetate, as shown in Fig. 3a, another three thioesterase genes (*fadM*, *tesB*, and *ybgC*) were proved to be effective on acetate production. The relative acetate titers increased by 76%, 348%, and 42%, respectively. This is not demonstrated through gene deletion, which may due to the similar function of other genes. With respect to butyrate, four genes *tesB*, *entH*, *menI*, and *yigI* were found to contribute to butyrate formation (Fig. 3b). Among the four genes, *entH* was also identified through gene deletion suggesting it is indeed an important thioesterase gene related to SCFAs formation.

### Investigation of the contribution of seven candidate genes to lactate production through gene deletion and overexpression

**Fig. 4:**
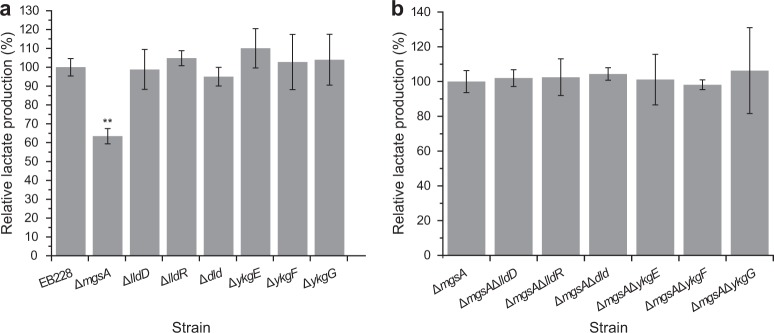
Lactate production after the first- and the second-round of gene deletions in *E. coli*. **a** first-round deletion, the lactate titer of strain EB228 was designated as 100%; **b** second-round deletion, the lactate titer of strain EB228Δ*mgsA* was designated as 100%. The data represent the means ± s.d. from three biological replicates. *Statistically significant difference with *P* < 0.05 and **Statistically significant difference with *P* < 0.01

As described above, a deletion of the *ldhA* gene did not abrogate lactate production. Similar to the SCFA-synthesis genes, we individually deleted seven candidate genes, which may be connected to lactate formation in strain EB228 using the Red-mediated recombination method.^29^ As shown in Fig. 4a, there were no significant changes in the lactate titer of any of the mutants except for strain EB228Δ*mgsA*, which showed a 37% decrease of the lactate titer (from 380 to 240 mg/L). To identify additional pathways for lactate production, a second-round of gene deletions was carried out based on strain EB228Δ*mgsA*. As shown in Fig. 4b, further knockouts of other genes in addition to the inactivation of *mgsA* had no significant effect on lactate production, suggesting that these six candidate genes did not contribute to lactate production in *E. coli*. All the mutants showed similar cell growth (Supplementary Fig. 3). Finally, a mutant strain with deletion of all seven candidate genes still produced detectable lactate. This result indicates that there are still unidentified pathways contributing to lactate production.

In addition to gene deletion, gene overexpression was also performed as described above. As *ykgE*, *ykgF*, and *ykgG* belong to a gene cluster, we cloned them into one vector. Cell growth of the total five strains was different making lactate titers vary accordingly (Supplementary Fig. 2b). Again, lactate titer/OD_600_ values were used to compare the contribution to lactate production of each gene. Among the tested four genes, only *lldD* showed positive effect on lactate production while the others did not work (Fig. 3c). In conclusion, we identified another two genes (*mgsA* and *lldD*) related to lactate metabolism in *E. coli* besides *ldhA*.

### Distribution of the selected functional genes in human gut microbiota

After testing the effects of nineteen candidate genes through gene deletion and overexpression, we identified six genes contributing to acetate production, eight genes contributing to butyrate production and two genes contributing to lactate production. Further investigation of their distribution in human gut microbiota was implemented. The bacteria were selected from the Human Microbiome Project database.^30,31^ As for acetate-synthesis genes, almost all of the listed bacteria contain the well-known *pta-ackA* genes (Table 3). Although the protein identities are relatively low, there is at least one gene coding acetate-related thioesterase among all the listed bacteria. This indicating the flexible metabolism of acetate. In bacterium *Megamonas hypermegale*, *pta-ackA* is not available, which means thioesterase gene is very likely to function instead. For butyrate-synthesis genes, only few bacteria contain the well-known *ptb-buk* genes suggesting butyrate is mainly produced from butyryl-CoA through thioesterase. Similar as acetate, there is also at least one gene coding butyrate-related thioesterase among all the listed bacteria (Table 3). In that case, these thioesterase genes may be used as markers for more butyrate-producing bacteria discovery.

**Table 3:** Distribution of well-known SCFA-synthesis genes and nine other functional genes in human gut microbiota

	*pta-ackA* ^a^	*ptb-buk* ^b^	*entH* ^b^	*fadM* ^a^	*menI* ^a,b^	*tesA* ^a,b^	*tesB* ^a,b^	*ybgC* ^a,b^	*ybhC* ^b^	*yciA* ^a,b^	*yigI* ^b^
*Acinetobacter radioresistens*	Y	N	20.00	21.01	21.38	Y	Y	Y	N	Y	31.36
*Bacteroides caccae*	Y	Y	10.98	21.90	14.97	10.08	11.15	23.70	N	15.57	14.37
*Bacteroides fragilis*	Y	Y	N	N	17.24	10.08	12.24	16.43	N	18.24	16.67
*Bifidobacterium breve*	Y	N	N	N	N	N	Y	N	N	N	N
*Bifidobacterium pseudocatenulatum*	Y	N	N	N	N	N	Y	N	N	N	N
*Clostridium bolteae*	Y	Y	18.75	10.14	15.28	N	13.89	13.04	N	15.60	19.87
*Coprococcus catus*	Y	N	18.75	15.94	20.55	12.82	11.15	14.93	N	14.39	17.42
*Desulfovibrio piger*	Y	N	18.31	19.18	19.01	N	N	24.29	N	13.33	20.50
*Enterobacter cancerogenus*	Y	N	Y	Y	78.68	Y	Y	Y	78.69	Y	20.12
*Enterococcus faecalis*	Y	Y	11.43	10.67	15.44	N	12.15	11.85	11.82	16.57	13.11
*Eubacterium hallii*	Y	N	12.65	11.43	14.72	12.35	12.20	11.03	N	20.96	14.11
*Faecalibacterium prausnitzii*	Y	N	16.67	15.00	14.39	12.00	13.24	17.04	N	13.87	13.38
*Fusobacterium ulcerans*	Y	N	22.38	N	23.78	N	N	N	N	N	N
*Helicobacter pylori*	Y	N	13.42	22.30	14.29	N	N	31.47	N	16.67	13.55
*Lactobacillus acidophilus*	Y	N	N	N	N	15.18	10.84	N	N	N	N
*Lactobacillus brevis*	Y	N	N	N	N	N	11.54	N	N	N	N
*Lactobacillus rhamnosus*	Y	N	N	N	N	Y	N	N	13.05	N	10.53
*Megamonas hypermegale*	N	N	N	N	N	13.55	N	N	N	N	13.09
*Methanobrevibacter smithii*	Y	N	16.55	17.02	N	N	N	16.55	N	18.18	12.26
*Oxalobacter formigenes*	Y	N	14.56	12.24	13.87	N	N	Y	N	15.44	15.38
*Parvimonas micra*	Y	N	N	N	N	N	10.64	N	10.70	N	N
*Providencia alcalifaciens*	Y	N	20.81	Y	19.46	Y	Y	Y	N	Y	15.48
*Ruminococcus* sp.	Y	N	N	N	N	11.06	11.07	N	N	N	N
*Ruminococcus torques*	Y	N	N	N	N	14.41	15.03	N	N	N	N
*Weissella paramesenteroides*	Y	N	18.44	N	21.99	10.53	10.14	11.94	N	N	16.98

Numbers in the table indicate the corresponding protein identity (%) with *E. coli*

Y: Existing annotated gene of the same name (gene name or enzyme name)

N: Gene does not exist or the corresponding protein identity is below 10%

^a^Genes involved in acetate production

^b^Genes involved in butyrate production

With respect to lactate, some bacteria have well-known *ldhA* gene for d-lactate production while some have *lldD* for l-lactate production (Table 4). Unexpectedly, we found many bacteria contain *mgsA* gene encoding methylglyoxal synthase. This methylglyoxal synthase involved pathway may contribute to lactate production second only to lactate dehydrogenase involved one. In conclusion, the discovered functional genes are widely distributed in gut microbiota.

**Table 4:** Distribution of well-known lactate-synthesis gene and two other functional genes in human gut microbiota

	*ldhA*	*mgsA*	*lldD*
*Acinetobacter radioresistens*	Y	N	Y
*Bacteroides caccae*	Y	Y	Y
*Bacteroides fragilis*	Y	Y	Y
*Bifidobacterium breve*	N	N	Y
*Bifidobacterium pseudocatenulatum*	N	N	Y
*Clostridium bolteae*	N	Y	Y
*Coprococcus catus*	N	Y	Y
*Desulfovibrio piger*	N	N	Y
*Enterobacter cancerogenus*	Y	Y	Y
*Enterococcus faecalis*	N	Y	Y
*Eubacterium hallii*	N	Y	Y
*Faecalibacterium prausnitzii*	N	Y	Y
*Fusobacterium ulcerans*	Y	Y	Y
*Helicobacter pylori*	Y	N	Y
*Lactobacillus acidophilus*	Y	N	Y
*Lactobacillus brevis*	Y	N	Y
*Lactobacillus rhamnosus*	Y	N	Y
*Megamonas hypermegale*	N	Y	Y
*Methanobrevibacter smithii*	N	N	Y
*Oxalobacter formigenes*	N	N	N
*Parvimonas micra*	N	N	N
*Providencia alcalifaciens*	Y	Y	N
*Ruminococcus* sp.	Y	Y	Y
*Ruminococcus torques*	N	Y	Y
*Weissella paramesenteroides*	Y	N	Y

Y: Existing annotated gene of the same name (gene name or enzyme name)

N: Gene does not exist or the corresponding protein identity is below 10%

## Discussion

**Fig. 5:**
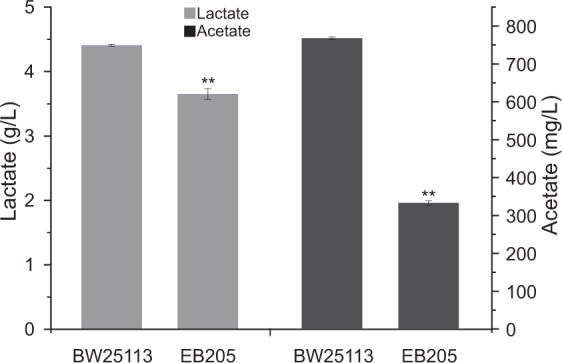
Acetate and lactate production in wild-type (BW25113) and mutant (EB205) *E. coli*. In the mutant, *pta-ackA* and *ldhA* were deleted. The data represent the means ± s.d. from three biological replicates. *Statistically significant difference with *P* < 0.05 and **Statistically significant difference with *P* < 0.01

Microbiota play important roles in the internal environment and health of humans and livestock. SCFAs and lactate are primary metabolites that have been implicated in the maintenance of a healthy intestinal physiology. It is well known that acetate can be produced from acetyl-CoA by phosphate acetyltransferase and acetate kinase (Pta-AckA), while lactate can be produced from pyruvate by lactate dehydrogenase (LDH). Wild-type *E. coli* produced 770 mg/L acetate and 4.41 g/L lactate. However, when the relevant genes were knocked out (*pta-ackA* for SCFAs and *ldhA* for lactate), the mutant strain still produced 330 mg/L acetate and 3.65 g/L lactate (Fig. 5). This implied that the well-known acetate and lactate pathways contributed only 57% and 17% to the acetate and lactate production, respectively.

To search for more pathways and enzymes involved, we selected twelve candidate genes for SCFA and seven genes for lactate synthesis based on genome database mining and literature searches. Based on gene deletion and overexpression, we identified six genes contributing to acetate production, eight genes contributing to butyrate production, and two genes contributing to lactate production. An investigation of the distribution of these functional genes in human gut microbiota indicated that most bacteria contain genes homologous to the well-known SCFAs (*pta*-*ackA*) and lactate (lactate dehydrogenase-encoding gene) biosynthesis genes of *E. coli*. Interestingly, some bacteria do not have the well-known genes but nevertheless contained possible alternative candidates. For example, in *M. hypermegale*, there is no *pta-ackA* gene but there are thioesterase genes (Table 3). Moreover, in many bacteria, the main lactate dehydrogenase gene *ldhA* does not exist. Instead, other genes, such as *mgsA* and/or *lldD*, were found. In *Oxalobacter formigenes* and *Parvimonas micra*, *ldhA*, *lldD*, and *mgsA* genes are all absent (Table 4). These untypical genotypes and corresponding phenotypes in gut microbiota are of particular interests.

As described in previous studies, SCFA-producing bacteria are very attractive due to the health benefits of SCFAs.^32–34^ SCFAs are rapidly adsorbed from the intestinal lumen and have different fates.^2^ Intracellular butyrate downregulate proinflammatory responses by inhibiting the activity of histone deacetylases.^2,14^ Unabsorbed butyrate is reused as an energy source.^6^ Acetate is reported to drive insulin secretion via parasympathetic inputs,^35^ promotes intestinal IgA response to microbiota,^36^ and has other positive effects on health.^2,3,6^ In this study, we tested the effects of the twelve selected candidate genes on SCFA production using *E. coli* as a model. New genes such as *fadM*, *menI*, *tesA*, *tesB*, *ybgC*, and *yciA* were identified functional. These genes can be used to study the complex metabolism of gut microbiota and to find more acetate-producing bacteria without *pta-ackA*.

With respect to butyrate, ten of the twelve selected gene knockouts reduced butyrate production. Interestingly, the sum of the production percentages of the ten genes was greater than 100%, indicating that some of the ten genes may have complementary functions in the synthesis of butyrate. Among the ten genes, *yciA* was most efficient in butyrate production. Further double knockouts of *yciA*-*tesB* and *yciA*-*paaI* greatly decreased the butyrate titer, by 92% and 85% of that the starting strain, respectively. These genes were also distributed in some gut bacteria, such as *Acinetobacter radioresistens*, *Enterobacter cancerogenus*, and *Providencia alcalifaciens*. Additionally, we discovered a thioesterase (encoded by *yciA*), which has different affinity for acetyl-CoA and butyryl-CoA. By manipulating the expression level of YciA, this enzyme may be a target for changing the proportion of SCFAs produced by gut microbiota. Based on the *yciA*-mutant, we found that variation of the SCFA titer affects the cell growth (Supplementary Fig. 1). This phenomenon indicates that the production of SCFAs may regulate the abundance of different microorganisms. Combine with gene overexpression results, *entH*, *menI*, *tesA*, *tesB*, *ybgC*, *ybhC*, *yciA*, and *yigI* were identified functional. This provides a basis for understanding the butyrate metabolism in gut microbiota.

Lactate is a very common metabolite in the human body.^37^ It is balanced in skeletal muscle and is an important metabolic substrate for the myocardium.^37,38^ Lactate links glycolytic metabolism and oxidative metabolism as a shuttle.^39^ It is involved in many biological processes and plays important role. For example, in gut, lactate can function as a signaling molecule and downregulate pro-inflammatory responses in intestinal epithelial and myeloid cells.^14,39^ It can accelerate intestinal stem-cell-mediated epithelial development and protect host from chemotherapy- and radiation-induced gut damage.^40^ Lactate was also found to have positive effect on resuscitation and treatment of injuries and illnesses.^39^ On the other hand, lactate was found to have a negative influence on human health and cell metabolism under certain conditions.^41^ Bouzier et al. found that lactate is the preferential carbon source of tumor cells in the presence of both lactate and glucose.^42^ Lactate promotes glutamine uptake and metabolism in oxidative cancer cells, while the produced glutamine is another metabolic substrate of cancer cells.^43^ The important role of lactate is continuous updating as the relevant studies continue. Moreover, precise editing of the gut microbiota was proved to be possible recently.^44^ Therefore, lactate-related genes may provide potential targets for this technique. In *E. coli*, we demonstrated that *mgsA* and *lldD* can contribute to lactate production in addition to *ldhA* (Fig. 1). This provides more gene targets for lactate rewiring. Notably, the mutant with a double knockout of *ldhA* and *mgsA* or even a combination of seven candidate genes knockout still produced lactate, which meant that there are other, still unknown contributing enzymes. Interestingly, some bacteria contain SCFA-producing enzymes but lack the key enzymes for lactate production (LDH and MgsA). Examples of such bacteria include *O. formigenes* and *P. micra*, which are of interests for further study.

## Methods

### Strains, plasmids, and primers

*E. coli* EB228 was constructed in our previous work.^45^ The well-known genes involved in acetate and lactate synthesis, *pta-ackA*, and *ldhA*, were both knocked out. An exogenous butanol pathway was introduced to maintain the redox balance so that the pathways of other fermentative acids can be easily removed. All strains and plasmids used in this study are listed in Supplementary Table 1. Primers are listed in Supplementary Table 2.

### DNA manipulation

Primers were synthesized by Invitrogen (Life Technologies, Beijing, China) followed by purification via polyacrylamide gel electrophoresis. Standard methods were used for plasmid and DNA fragment electroporation.^46^ PCR products and DNA fragments were purified using the E.Z.N.A Cycle Pure Kit (Omega Biotek Inc., Guangzhou, China). The chromosomal gene deletion was performed following previously described procedures.^29^ DNA sequencing services were provided by TSINGKE (TSINGKE biological technology Co., Ltd., Beijing, China).

### Strain development

Based on strain EB228, a series of mutant strains were constructed using the Red-mediated one-step inactivation method.^29^ Taking Δ*lldD*::FRT as an example, primers 19–20 (*lldD*-KoF, *lldD*-KoR) were used to amplify the FRT-*kan*-FRT fragment with short homologous sequences on both sides. Next, the fragment was electroporated into strain EB228 carrying the plasmid pKD46. Under the action of Red recombinase, the *lldD* gene was replaced by the FRT-*kan*-FRT fragment in the mutant, which was selected on agar plates with kanamycin and verified using primers 21−22 (*lldD*-F, *lldD*-R). A second replacement was achieved using plasmid pCP20, which can remove the FRT-*kan*-FRT fragment bringing one FRT into the locus. Finally, strain EB228Δ*lldD* was successfully constructed. For gene overexpression, a pACYC184-derived plasmid pAC2 containing a constitutive promoter miniPtac was used. Gibson assembly reagent (New England BioLabs, Beijing, China) was used for plasmids construction. All the EB228-derived mutant strains are listed in Supplementary Table 1.

### Growth and fermentation conditions

*E. coli* cells were grown aerobically overnight in Luria-Bertani (LB) medium at 37 °C in a rotary shaker (200 rpm). Unless otherwise indicated, 5% of the culture was transferred into M9Y medium^28^ containing glucose for oxygen-limited fermentation. Tube fermentation was conducted in a 37 °C incubator using 10 mL M9Y medium containing 2% (w/v) glucose in a half-sealed 15 mL tube (BD Biosciences, San Jose, CA). The fermentation process lasted for 72 h. All the experiments were performed with three repeats.

### Enzyme assay

Thioesterase activity of the strains EB228 and EB228Δ*yciA* was determined based on a published method.^47^ Log-phase cells were collected and lysed by sonication to obtain a crude extract. The absorbance of 5-thio-2-nitrobenzoate at 412 nm was measured after the reaction of 5,5′-dithiobis-(2-nitrobenzoic acid) (DTNB) with CoA. The standard assay mixture in a total volume of 200 μL contained 1 mM DTNB, 0.2 M KCl, 50 mM K^+^-HEPES (pH 7.5) and 0.01 mM acetyl-CoA or butyryl-CoA (Promega, Beijing, China). The reaction was started by the addition of the crude extract and was carried out at 25 °C. The molar extinction coefficient of 5-thio-2-nitrobenzoate (*ε* = 13.6 mM^−1^ cm^−1^) was used to calculate the relative enzyme activity.

### Analytical methods

All the nineteen candidate genes’ sequences were obtained on KEGG database. DNAMAN software (Lynnon LLC., CA) was used for protein sequence alignment. Cell density was determined by a UV-visible spectrophotometer (UV-2802PC; Unico, Shanghai, China) via the optical density at 600 nm. After 72 h fermentation, the concentrations of sugars and organic acids concentrations in the samples were analyzed using an Agilent 1260 high performance liquid chromatography (HPLC) system (Agilent Technologies, Santa Clara, CA) equipped with a Bio-Rad HPX-87H column (Bio-Rad Laboratories, Inc., Richmond, CA) with 5 mM H_2_SO_4_ as the mobile phase (10 μL injection, 0.5 mL/min, 15 °C). Signals were detected by a refractive index (RI) detector.

### Statistical analysis

The statistical analysis of the data was performed using Student’s *t*-test in Origin software (OriginLab, MA) where appropriate. *P* values of <0.05 were considered to indicate statistical significance. *P < 0.05; **P < 0.01.

### Reporting summary

Further information on research design is available in the Nature Research Reporting Summary linked to this article.

## Supplementary information

Supplementary Information

Reporting Summary Checklist

## Data Availability

All data supporting the findings of this study are available from the corresponding author upon reasonable request.

## Supplementary information

**Supplementary information** accompanies the paper on the *npj Biofilms and Microbiomes* website (10.1038/s41522-019-0092-7).
