# Supplementary material for: Discovery of potential genes contributing to the biosynthesis of short-chain fatty acids and lactate in gut microbiota from systematic investigation in E. coli
Source: NPJ Biofilms Microbiomes. 2019 Jul 12;5:19. doi: 10.1038/s41522-019-0092-7 (PMC6626047; doi:10.1038/s41522-019-0092-7)
Supplement: Supplementary file 1 — Supplementary Information [file 41522_2019_92_MOESM1_ESM.pdf]

Supplementary Information for "Discovery of potential genes contributing to the biosynthesis of short-chain fatty acids and lactate in gut microbiota from systematic investigation in *E. coli*"

Chunhua Zhao[1,2], Hongjun Dong[1,3], Yanping Zhang[1*] and Yin Li[1*]

[1]CAS Key Laboratory of Microbial Physiological and Metabolic Engineering, State Key Laboratory of Microbial Resources, Institute of Microbiology, Chinese Academy of Sciences, Beijing, China

[2]University of Chinese Academy of Sciences, Beijing, China

[3]Present address: Department of Chemistry, University of California, Berkeley, Berkeley, California, USA

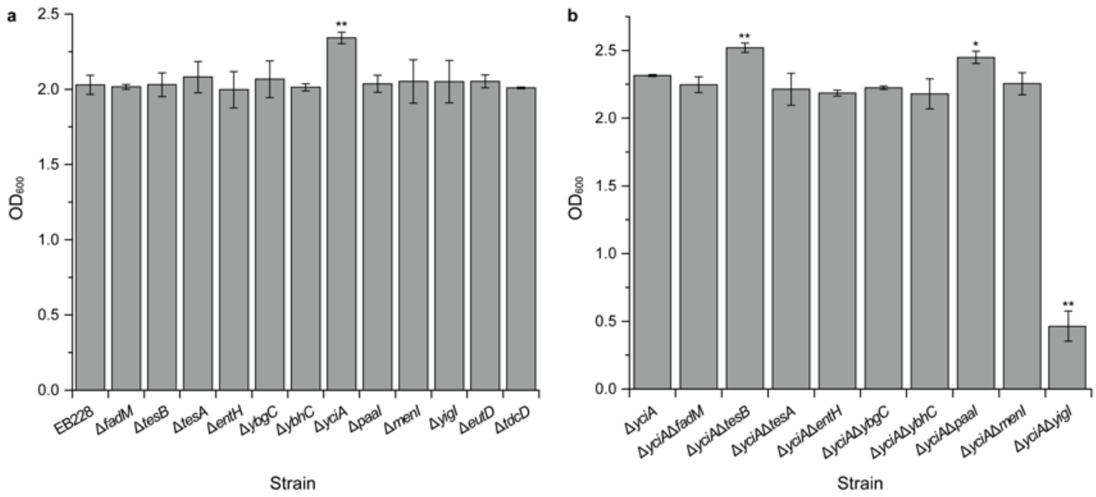

Supplementary Figure 1 The final OD_{600} values of *E. coli* strains after first- and second-round of SCFAs related gene deletions. a, first-round deletion; b, second-round deletion. The data represent the means ± s.d. from three biological replicates.

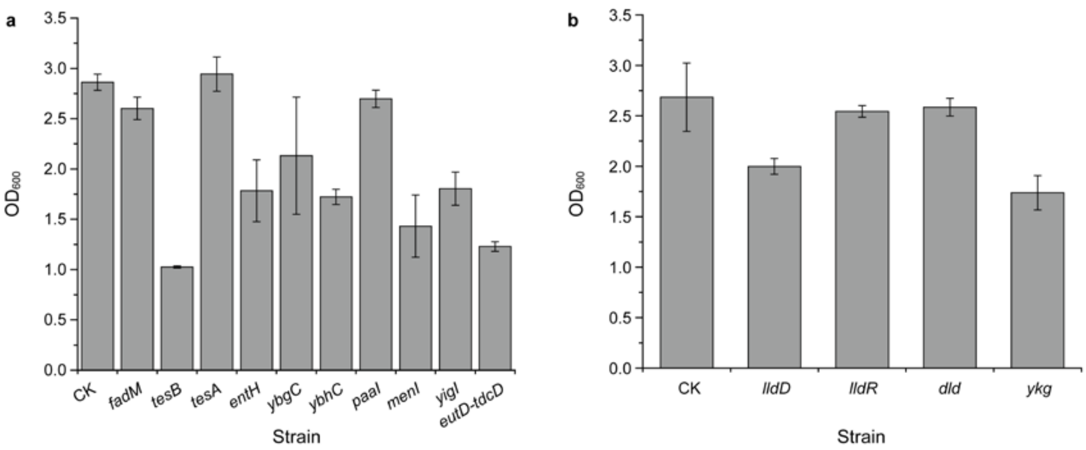

Supplementary Figure 2 The final OD_{600} values of *E. coli* strains after overexpressing SCFAs-related genes and lactate-related genes. a, overexpressing SCFAs-related genes. CK: EB228Δ*yciA*,pAC2; Gene name indicating strain with overexpressed corresponding gene. For instance, *fadM* indicating EB228Δ*yciA*,pAC2-*fadM*. b, overexpressing lactate-related genes. CK: EB228Δ*mgsA*,pAC2; Gene name indicating strain with overexpressed corresponding gene. For instance, *lldD* indicating EB228Δ*mgsA*,pAC2-*lldD*. The data represent the means ± s.d. from three biological replicates.

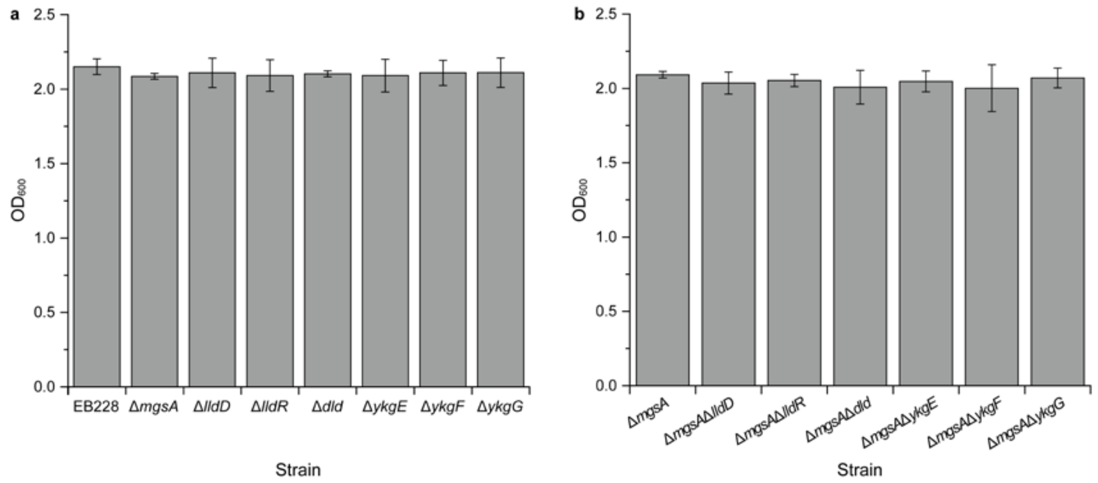

Supplementary Figure 3 The final OD_{600} values of *E. coli* strains after first- and second-round of lactate related gene deletions. a, first-round deletion; b, second-round deletion. The data represent the means ± s.d. from three biological replicates.

Supplementary Table 1 Strains and plasmids used in this study

Strain or plasmid		Characteristics	References or sources
Strain	EB222	Butanol-producing strain constructed based on BW25113	ref. [1]
	EB228	Derived from EB222, Δ*maeB*::*fdh*, Δ*mdh*::*fdh*	This study
	EB228Δ*mgsA*	Derived from EB228, Δ*mgsA*::FRT	This study
	EB228Δ*lldD*	Derived from EB228, Δ*lldD*::FRT	This study
	EB228Δ*lldR*	Derived from EB228, Δ*lldR*::FRT	This study
	EB228Δ*dld*	Derived from EB228, Δ*dld*::FRT	This study
	EB228Δ*ykgE*	Derived from EB228, Δ*ykgE*::FRT	This study
	EB228Δ*ykgF*	Derived from EB228, Δ*ykgF*::FRT	This study
	EB228Δ*ykgG*	Derived from EB228, Δ*ykgG*::FRT	This study
	EB228Δ*fadM*	Derived from EB228, Δ*fadM*::FRT	This study
	EB228Δ*tesB*	Derived from EB228, Δ*tesB*::FRT	This study
	EB228Δ*tesA*	Derived from EB228, Δ*tesA*::FRT	This study
	EB228Δ*entH*	Derived from EB228, Δ*entH*::FRT	This study
	EB228Δ*ybgC*	Derived from EB228, Δ*ybgC*::FRT	This study
	EB228Δ*ybhC*	Derived from EB228, Δ*ybhC*::FRT	This study
	EB228Δ*yciA*	Derived from EB228, Δ*yciA*::FRT	This study
	EB228Δ*paaI*	Derived from EB228, Δ*paaI*::FRT	This study
	EB228Δ*menI*	Derived from EB228, Δ*menI*::FRT	This study
	EB228Δ*yigI*	Derived from EB228, Δ*yigI*::FRT	This study
	EB228Δ*yciA*Δ*fadM*	Derived from EB228, Δ*yciA*::FRT, Δ*fadM*::FRT	This study
	EB228Δ*yciA*Δ*tesB*	Derived from EB228, Δ*yciA*::FRT, Δ*tesB*::FRT	This study
	EB228Δ*yciA*Δ*tesA*	Derived from EB228, Δ*yciA*::FRT, Δ*tesA*::FRT	This study

	EB228Δ*yciA*Δ*entH*	Derived from EB228, Δ*yciA*::FRT, Δ*entH*::FRT	This study
	EB228Δ*yciA*Δ*ybgC*	Derived from EB228, Δ*yciA*::FRT, Δ*ybgC*::FRT	This study
	EB228Δ*yciA*Δ*ybhC*	Derived from EB228, Δ*yciA*::FRT, Δ*ybhC*::FRT	This study
	EB228Δ*yciA*Δ*paaI*	Derived from EB228, Δ*yciA*::FRT, Δ*paaI*::FRT	This study
	EB228Δ*yciA*Δ*menI*	Derived from EB228, Δ*yciA*::FRT, Δ*menI*::FRT	This study
	EB228Δ*mgsA*Δ*yciA*Δ*tesB*	Derived from EB228, Δ*mgsA*::FRT, Δ*yciA*::FRT, Δ*tesB*::FRT	This study
Plasmid	pKD4	*bla*, FRT, *kan*	ref. [2]
	pKD46	*bla, araC, gam-bet-exo*	ref. [2]
	pCP20	*bla, flp, cat*	ref. [2]
	pAC2	Derived from pACYC184, miniPtac, *cat*	This study
	pAC2-*fadM*	Derived from pAC2, *fadM*	This study
	pAC2-*tesB*	Derived from pAC2, *tesB*	This study
	pAC2-*tesA*	Derived from pAC2, *tesA*	This study
	pAC2-*entH*	Derived from pAC2, *entH*	This study
	pAC2-*ybgC*	Derived from pAC2, *ybgC*	This study
	pAC2-*ybhC*	Derived from pAC2, *ybhC*	This study
	pAC2-*paaI*	Derived from pAC2, *paaI*	This study
	pAC2-*menI*	Derived from pAC2, *menI*	This study
	pAC2-*yigI*	Derived from pAC2, *yigI*	This study
	pAC2-*eutD-tdcD*	Derived from pAC2, *eutD-tdcD*	This study
	pAC2-*lldD*	Derived from pAC2, *lldD*	This study
	pAC2-*lldR*	Derived from pAC2, *lldR*	This study
	pAC2-*dld*	Derived from pAC2, *dld*	This study
	pAC2-*ykg*	Derived from pAC2, *ykgEFG*	This study

bla, ampicillin resistance gene; *kan*, kanamycin resistance gene; *gam-bet-exo*, Red recombinase genes; *flp*, flippase (Flp recombinase) gene; *cat*, chloramphenicol resistance gene; FRT, flippase recognition target.

Supplementary Table 2 Primers used in this study

Primer number	Primer name	Sequence (5′-3′)
1	*mgsA*-KoF	CATATTGCGCTGGTGGCACACGATCACTGCAAACAAATGCTGTAG GCTGGAGCTGCTTC
2	*mgsA*-KoR	GATCAGAATATCGACCGCGTCGTTGAAATGCGGCGACTGGTGGG AATTAGCCATGGTCC
3	*mgsA*-F	ATGGAACTGACGACTCGCAC
4	*mgsA*-R	TTACTTCAGACGGTCCGCGAG
5	*lldD*-KoF	AAGTGGCGCTGCGCCAGCGTATTCTGAAAAACATGTCCGATGTAG GCTGGAGCTGCTTC
6	*lldD*-KoR	CGCTGATCGATTTCGCGCCAGTCAGCGTCATCGCCACTTTTGGGA ATTAGCCATGGTCC
7	*lldD*-F	ATGATTATTTCCGCAGCCAGC
8	*lldD*-R	CTATGCCGCATTCCCTTTCGC
9	*lldR*-KoF	ATGATTGTTTTACCCAGACGCCTGTCAGACGAGGTTGCCGTGTAG GCTGGAGCTGCTTC
10	*lldR*-KoR	TCATGCGTTTTTCTCCCTCGAATGCTCATTATGCTCACCGTGGGAA TTAGCCATGGTCC
11	*lldR*-F	GTATTCCTGACCGGGTCGGATAC
12	*lldR*-R	CCGCGATCGCGCAGTACATAAAG
13	*dld*-KoF	TCAAACCGCTGGGACGCGAACCGCACTCAGTGATTGGATCTGTA GGCTGGAGCTGCTTC
14	*dld*-KoR	GAATGTCTTCGACTTCATCGGAATGCACCGCCTGATAACGTGGGA ATTAGCCATGGTCC
15	*dld*-F	ATGTCTTCCATGACAACAACTG
16	*dld*-R	TTACTCCACTTCCTGCCAGTT
17	*ykgE*-KoF	GTGAATGTCAATTTCTTTGTCACCTGTATTGGTGACGCCCTGTAGG CTGGAGCTGCTTC
18	*ykgE*-KoR	TCAGCGGCTCATCAACACTTCAGCAATATGCATCACTTTGTGGGA

		ATTAGCCATGGTCC
19	*ykgE*-F	ATATCTCAATAACCCCTGAATA
20	*ykgE*-R	GCCAGCATCCTGCAACACATG
21	*ykgF*-KoF	AATTGGGGCACTGGGAGGAGTGGCGCGATCGGGCCGCCCATGTA GGCTGGAGCTGCTTC
22	*ykgF*-KoR	CACAAGCTGTGCATAAAGAGCAGGCGTAGGGTAAATCTTTTGGG AATTAGCCATGGTCC
23	*ykgF*-F	ATGTCGATCAAAACCAGTAATA
24	*ykgF*-R	TTATCCATTCTTTTTCTCCTG
25	*ykgG*-KoF	ATGGATAATCGAGGCGAATTTTTGAATAACGTTGCTCAGGTGTAG GCTGGAGCTGCTTC
26	*ykgG*-KoR	TCAACAATCCTCAATAATCAGATACACCGCTTTCACCGGGTGGGA ATTAGCCATGGTCC
27	*ykgG*-F	TGCGTATTCCGCTGTCAAAAC
28	*ykgG*-R	GTGATGGCTGTGTAACTATAAG
29	*fadM*-KoF	GACGTTTACCAGCACGTCAACAACGCCCGCTACCTTGAATTGTAG GCTGGAGCTGCTTC
30	*fadM*-KoR	CTTCCAGAGCTAATGCTTTCTGCGTTTTAAGATCAATACATGGGAA TTAGCCATGGTCC
31	*fadM*-F	ACACAAATCAAAGTTCGTGGATATCATC
32	*fadM*-R	CCATCTGCTCCAGCTTTTCGCGCAATTC
33	*tesB*-KoF	GTGAAGATTTAGGTTTACGCCAGGTGTTTGGCGGCCAGGTTGTAG GCTGGAGCTGCTTC
34	*tesB*-KoR	GTATAAAACTCACCGCGCACAAAGCCACGTGCGCTGGACGTGGG AATTAGCCATGGTCC
35	*tesB*-F	AAATTTACTGACATTGTTAAATCTGG
36	*tesB*-R	ATTACGCATCACCCCTTCCTGAACGG
37	*tesA*-KoF	CAGCGGACACGTTATTGATTCTGGGTGATAGCCTGAGCGCTGTAG GCTGGAGCTGCTTC
38	*tesA*-KoR	TTGGGATGAATACCGTCATCCTGCATCCATTGTGGCTTGATGGGAA

		TTAGCCATGGTCC
39	*tesA*-F	TGTTTTCCGCTGGCATTTGCCCTTCCTG
40	*tesA*-R	AGGCTGCAACTGCTTCGCCATCCAGTCG
41	*entH*-KoF	AACGCCACCAGCGATAACACAATGGTGGCGCATCTGGGAATGTA GGCTGGAGCTGCTTC
42	*entH*-KoR	CAGCAACGCCGCCCCTGTTCATCGAAAACGACGATTTCCCTGGG AATTAGCCATGGTCC
43	*entH*-F	ATCTGGAAACGCCATTTAACGCTCGACG
44	*entH*-R	CAAAACTGCCGTACCCAGCCGACAAGTG
45	*ybgC*-KoF	TACTATGAAGATACCGATGCCGGTGGTGTGGTGTACCACGTGTAG GCTGGAGCTGCTTC
46	*ybgC*-KoR	GCGCACGAGGCTTCATTTTGAGTGGGTCAACGCAAACAACTGGG AATTAGCCATGGTCC
47	*ybgC*-F	ATACAACGCTGTTTCGATGGCCGGTTCG
48	*ybgC*-R	CTGCTTAAACTCCGCGACAATAGACTTG
49	*ybhC*-KoF	GAATTTCGATGCTCAACACTATTTTGCATCCCTGACACCATGTAGG CTGGAGCTGCTTC
50	*ybhC*-KoR	AGAGATCACCGCATCGGCCCACGGTTTAGCCGTGTTAAAATGGG AATTAGCCATGGTCC
51	*ybhC*-F	CGCCCGATCAACGTCCTTCTGATCAAAC
52	*ybhC*-R	TAGTGTCATTCAGATTGCGCTGTATTTC
53	*yciA*-KoF	GTTTTACGTACTTTAGCCATGCCCGCCGATACCAATGCCATGTAGG CTGGAGCTGCTTC
54	*yciA*-KoR	ATCAACCGCGACATACTTAAATAATGCTTCTGTCGCTTTATGGGAA TTAGCCATGGTCC
55	*yciA*-F	GTCTACAACACATAACGTCCCTCAGG
56	*yciA*-R	CTCAACAGGTAAGGCGCGAGGTTTTC
57	*paaI*-KoF	TATGAGAACGATGCCTGCGCCAAAGCGCTTGGCATCGACATGTAG GCTGGAGCTGCTTC
58	*paaI*-KoR	CCGCGAAACAGCGCAACCGTTTTTTGTTGTTGGTTAACAATGGGA

		ATTAGCCATGGTCC
59	*paaI*-F	GTCATAAGGCCTGGCAAAATGCCCATGC
60	*paaI*-R	GGCTTCTCCTGTAATGGTGCCGCCGATG
61	*menI*-KoF	AATGCTATGGGTGAAGGAAACATGGTGGGGTTCCTGGATATGTAG GCTGGAGCTGCTTC
62	*menI*-KoR	AACGCCCTTTCTCATCGAAGATTTCAATCTGCCAGACCTGTGGGA ATTAGCCATGGTCC
63	*menI*-F	TATGGAAACGGAAAATCACCCTGGAAGC
64	*menI*-R	AAAATGGCGGTCGTCAATCGTGACGAAC
65	*yigI*-KoF	TATCACATGCCATTTAACCGCGCATTGGGGATGGAACTGGTGTAG GCTGGAGCTGCTTC
66	*yigI*-KoR	TTCATTGTGTAATTCAACGCGGGCGACGGCGACTTTATTGTGGGA ATTAGCCATGGTCC
67	*yigI*-F	TGCCGTACTGACCGCTGAACAAGCCCTG
68	*yigI*-R	CCTACCATATAGGTGGCGGTGGCACTGG
69	*eutD*-KoF	ATGATTATTGAACGTTGTCGTGAACTGGCGTTGCGAGCGCTGTAG GCTGGAGCTGCTTC
70	*eutD*-KoR	TCATTCAACCAGTGTTTGTAAACTGCTTTCGCGGTTCACTTGGGA ATTAGCCATGGTCC
71	*eutD*-F	AATCTGCTACGCACCAAAGTG
72	*eutD*-R	GGCTTTACAGGCTGCAACATC
73	*tdcD*-KoF	GGCCTTAATTGGCCACCGCATCGCTCACGGCGGCAGTATTTGTAG GCTGGAGCTGCTTC
74	*tdcD*-KoR	CCATCCAGGCGACGTAATGAAGCTGCGTGTCCGGCAATATTGGGA ATTAGCCATGGTCC
75	*tdcD*-F	ATGAATGAATTTCCGGTTGTT
76	*tdcD*-R	TTATGCAAATTCTGCGGGCGC

Supplementary References

1. Zhao, C., Lin, Z., Dong, H., Zhang, Y. & Li, Y. Reexamination of the physiological role of PykA in *Escherichia coli* revealed that it negatively regulates the intracellular ATP levels under anaerobic conditions. *Appl Environ Microbiol* **83**, e00316-17 (2017).
2. Datsenko, K. A. & Wanner, B. L. One-step inactivation of chromosomal genes in *Escherichia coli* K-12 using PCR products. *Proc Natl Acad Sci USA* **97**, 6640-6645 (2000).
